# ^18^F-FDG PET radiomic analysis to predict outcomes in metastatic melanoma treated with immune checkpoint inhibitors

**DOI:** 10.3389/fimmu.2026.1642620

**Published:** 2026-02-25

**Authors:** Karim Amrane, Coline Le Meur, David Bourhis, Christian Berthou, Olivier Pradier, Laurent Misery, Delphine Legoupil, Maxime Etienne, Georges-Philippe Fontaine, Cyril Leleu, Romain Floch, Pierre-Yves Salaun, Ronan Abgral, Vincent Bourbonne

**Affiliations:** 1 Department of Oncology, Regional Hospital of Morlaix, Morlaix, France; 2 LBAI, UMR1227, Univ Brest, Inserm, Brest, France; 3 Department of Radiotherapy, University Hospital of Brest, Brest, France; 4 Department of Nuclear Medicine, University Hospital of Brest, Brest, France; 5 UMR Inserm 1304 GETBO, IFR 148, University of Western Brittany, Brest, France; 6 Department of Hematology, University Hospital of Brest, Brest, France; 7 Inserm, UMR1101, LaTIM, University of Western Brittany, Brest, France; 8 Department of Dermatology, University Hospital of Brest, Brest, France; 9 Laboratoire sur les Interactions Épithéliums-Neurones (LIEN-EA4685), University of Western Brittany, Brest, France; 10 Department of Dermatology Centre Hospitalier de Cornouaille, Quimper, France; 11 Nuclear Medicine Centre Georges Charpak, Quimper, France; 12 Department of Radiotherapy, Centre Hospitalier de Cornouaille, Quimper, France

**Keywords:** BRAF, FDG-PET, immune checkpoint inhibition, melanoma, NRAS, radiomic

## Abstract

**Purpose:**

Cutaneous melanoma (CM) incidence is rising, and despite advances in immune checkpoint inhibitors (ICI), many metastatic patients do not respond or develop resistance. This study aimed to evaluate the prognostic value of a pre-treatment FDG-PET/CT-based radiomic model (MEL-RAD) for predicting 1-year progression-free survival (1y-PFS) in metastatic CM patients treated with first-line ICI.

**Methods:**

We retrospectively included 154 metastatic CM patients from two centers who underwent pre-treatment FDG-PET/CT before ICI initiation. Patients were split into a development cohort (n=95) and an independent testing cohort (n=59). Radiomic features were extracted and harmonized to reduce inter-cohort variability. A two-step feature selection identified three key wavelet-transformed texture features used to build the MEL-RAD predictive model. The model’s performance was assessed by receiver operating characteristic (ROC) analysis, sensitivity, specificity, and predictive values. Survival analyses (progression-free (PFS) and overall survival (OS)) were performed with Cox regression and Kaplan-Meier methods.

**Results:**

In the development cohort, MEL-RAD achieved an AUC of 0.74 (p<0.0001) for predicting 1y-PFS. Using a 55% probability threshold, sensitivity was 93.8%, specificity 31.9%, with positive and negative predictive values of 58.4% and 83.4%, respectively. Patients with MEL-RAD >55% had significantly worse PFS (HR = 2.73, p=0.0009) and OS (HR = 3.20, p=0.0003). These results were externally validated: in the testing cohort, MEL-RAD positivity remained significantly associated with poorer PFS (HR = 2.73, p=0.047), and showed non-significant for OS.

**Conclusion:**

The MEL-RAD radiomic model based on pre-treatment FDG-PET/CT offers a non-invasive biomarker to stratify metastatic CM patients treated with immunotherapy.

## 1 Introduction

Cutaneous melanoma (CM) is a common cancer in developed countries, with increasing incidence rates (1). Early-stage melanoma has a high 5-year survival rate (98.4%), but survival decreases at advanced stages, with rates of 63.6% for regional nodal involvement and 22.5% for metastatic disease (2). Advances in targeted therapies and immunotherapies, especially immune checkpoint inhibitors (ICI) targeting CTLA-4 (e.g., ipilimumab) (3) and PD-1 (e.g., nivolumab, pembrolizumab), have improved survival (4–6). The 5-year survival rate for CM patients treated with nivolumab is 39% (5), and the combination of nivolumab and ipilimumab shows a 5-year survival rate of 52% (6). However, the 12-month progression-free survival (PFS) rate with nivolumab+ipilimumab is 47.7%, 40 to 65% of metastatic melanoma do not respond to ICI therapies, and among responders, 43% develop secondary resistance after 3 years (7). Current challenges to improve the prediction of treatment response in melanoma include the investigation of the tumor microenvironment and tumor heterogeneity through “omics” approaches, including genomics and radiomics (8, 9).

Pre-therapeutic ^18^F-fluorodeoxyglucose positron emission tomography-computed tomography (FDG-PET/CT) is commonly used to stage advanced CM (10, 11). Studies have shown that tumor uptake, assessed through quantitative [e.g., standardized uptake value (SUV)] and volumetric [e.g., metabolic tumor volume (MTV), total lesion glycolysis (TLG)] parameters, is a prognostic factor for survival in various cancers, including CM (12). However, no definitive cut-off value has been established to select patients with poor prognosis based on these imaging parameters.

Radiomic approaches extract textural features (TFs) from imaging modalities such as PET to characterize tumor heterogeneity, which is significant in CM, with regions of hypoxia, necrosis, high cell proliferation, and angiogenic variability (13). While first-order features with SUV, MTV, and TLG capture basic tumor characteristics, second- and higher-order features model spatial voxel relationships, providing a more comprehensive analysis of heterogeneity (14). This method could help personalize treatment for CM, a cancer with poor prognosis, by enhancing therapy effectiveness. However, challenges in reproducibility and PET calibration remain, though advancements in harmonized protocols, artificial intelligence, and deep learning are improving these techniques (15).

Accurate early identification of patients at high risk of progression under ICI remains a major unmet clinical need in metastatic melanoma, as treatment intensification or de-escalation strategies are currently based on limited predictive tools (16, 17).

The aim of this study is to evaluate the prognostic value of a pre-treatment FDG-PET/CT-based radiomic model (MEL-RAD) for disease progression in a large cohort of patients with metastatic CM treated with ICI.

## 2 Materials and methods

### 2.1 Patients

Consecutive patients with histologically confirmed CM, treated with first-line ICI at two different centers were retrospectively enrolled in this study between August 2015 and September 2023. All patients underwent pre-treatment FDG-PET/CT imaging and were recorded in the national prospective clinical cohort called Melbase (NCT02828202). The study adhered to the principles outlined in the Declaration of Helsinki and received approval from the French Advisory Committee on Information Processing in Health Research (CCTIRS). For patients still alive at the time of the study, written information about the research was provided to obtain their non-opposition for participation. For deceased patients, a waiver of information was obtained from the CCTIRS. This study is performed within the Melbase database, n° 12,027, 2012, favorable opinion *from Comité de Protection des Personnes Ile-de-France XI.* For this imaging-based study, inclusion was restricted to centers with full access to raw FDG-PET/CT data required for radiomic analysis, which limited the analysis to two participating centers.

Inclusion criteria were: (i) biopsy-proven metastatic or locally advanced unresectable CM; (ii) patients who received at least one dose of ICI as first-line treatment, including pembrolizumab (200 mg every 3 weeks IV), nivolumab (240 mg every 2 weeks or 480 mg every 4 weeks IV), or combination therapy with ipilimumab (3 mg/kg) + nivolumab (1 mg/kg) every 3 weeks for 4 cycles, followed by nivolumab maintenance (240 mg every 2 weeks or 480 mg every 4 weeks IV); (iii) no history of other primary malignancies; (iv) availability of pre-treatment FDG-PET/CT performed within 8 weeks prior to the first ICI infusion, according to the European Association of Nuclear Medicine (EANM) guidelines (18).

Exclusion criteria were as follows: (i) refusal to participate in the study, (ii) administration of ICI as second-line treatment, (iii) FDG-PET/CT performed outside the two participating centers, (iv) patients with only active brain metastasis and (v) patients with advanced primary cancers other than melanoma.

### 2.2 Follow-up

Clinical data collected included age, sex, Eastern Cooperative Oncology Group Performance Status (ECOG PS), corticosteroid use, AJCC staging (19), previous treatments, presence of metastases at diagnosis, baseline lactate dehydrogenase (LDH) levels and genetic mutations (*i.*e, BRAF, NRAS). Treatment decisions were made according to standard guidelines by a multidisciplinary melanoma board. Patients were clinically followed for at least one year to assess PFS and overall survival (OS), in accordance with National Comprehensive Cancer Network (NCCN) and European Society for Medical Oncology (ESMO) recommendations.

### 2.3 FDG PET/CT imaging

All imaging were performed at Center 1 and Center 2 using different PET systems, according to the EANM guidelines (18), whose characteristics are summarized in **Supplementary Table 1**.

Standard patient preparation consisted of a fast of at least 6 hours and a serum blood glucose level of <7 mmol/l. After an intravenous injection of FDG (detailed PET/CT acquisition, reconstruction parameters and scanner characteristics for each center provided in **Supplementary Table 1**), patients rested in a quiet room for approximately 60 minutes to allow for tracer uptake.

Patient were scanned supine arms along the body. First, a craniocaudal CT scan was performed, following an intravenous administration of iodinated contrast medium (1.5 ml/kg), unless contraindicated. Then, whole-body PET emission data were acquired in 3D mode, corrected for background noise, random events, and reconstructed with and without attenuation correction.

### 2.4 Images analysis and radiomics workflow

Patients from Center 1 served as the development cohort (training + internal validation) and patients from Center 2 served as the testing cohort.

All tumors were analyzed using MIM software (MIM Maestro^®^, Cleveland, OH, USA). Prior to analysis, each PET scan was rescaled, resulting in voxel sizes of 1 x 1 x 1 mm. Tumor lesions were automatically segmented using a method based on the PERCIST criteria for treatment response assessment, which delineates the target tumor with a threshold set of 1.5 x mean liver SUV within a 3-cm spherical volume of interest (VOI) in the right lobe + 2 SD (20). Manual corrections were made to correct for certain volume errors (brain, kidneys, bladder,…). Radiomic features of interest were then extracted using the PyRadiomics toolbox (21), with a fixed bin width of 0.1 and the application of 8 wavelet filters to the PET data. Images were processed using the full set of “coif1” wavelet decomposition sub-bands (LLL, LLH, LHL, HLL, HLH, HHL, and HHH), using the default values for start_level and level. For both the original images and their wavelet-transformed counterparts, features were extracted from the following classes: first-order statistics, shape, GLCM, GLRLM, GLSZM, GLDM, and NGTDM. The full list of features is provided as **Supplementary Table 2** (https://doi.org/10.5281/zenodo.15584144). This process resulted in the extraction of 856 features per patient (**Supplementary Table 3**).

Harmonization of the radiomic features was performed to account for potential differences between cohorts, imaging devices, and acquisition protocols. After normalization, the a posteriori neuroCombat procedure (22) was applied to all features, using the initial development cohort from Center 1 as a reference. Harmonization was performed to reduce scanner- and center-related variability while preserving biological inter-patient variability using the a posteriori neuroCombat algorithm, implemented in Python. This approach adjusts feature distributions across centers while maintaining associations with clinical outcomes.

**Figure 1:**
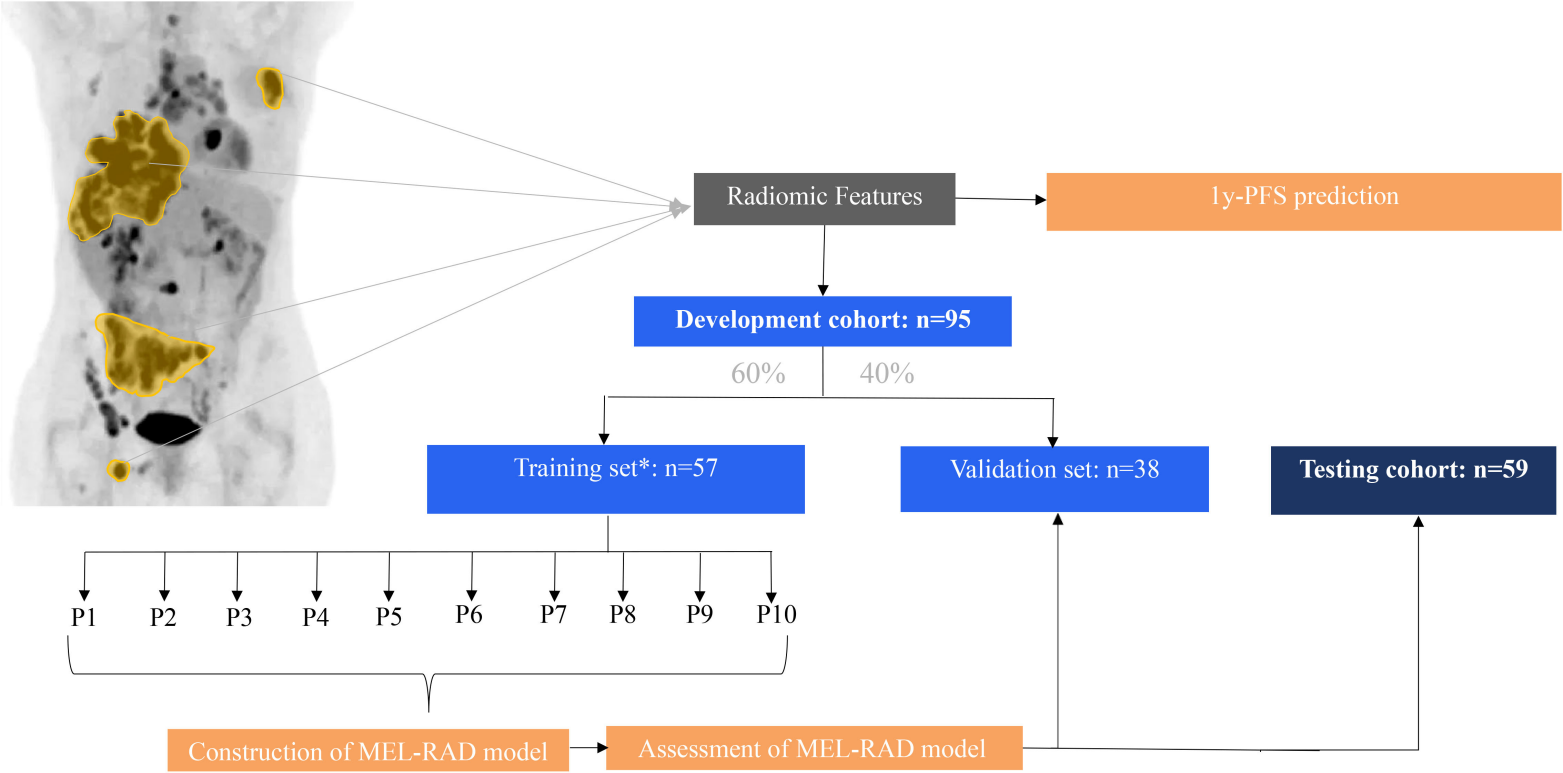
Workflow for the radiomics analysis. (1y-PFS, 1-year progression (including death) free status; *, 10-fold cross-validation).

These different steps are described in **Figure 1**.

### 2.5 Feature set reduction and model building

Radiomic features of interest were first selected in the development cohort using a previously developed and published two-step approach (23, 24). Briefly, only features that were significantly associated with 1year-progression (including death) free status (1y-PFS) based on the Mann-Whitney test were retained. Adjusted p-values were not applied at this exploratory feature selection stage, as redundancy was subsequently controlled through correlation filtering and external validation. Pair-wise correlations between the retained features was then assessed using the Spearman correlation coefficient, where only the most significant feature with a Spearman coefficient > 0.7 was retained. Once selected, the remaining features were processed through the model building workflow.

The development and internal validation of the prediction model was performed on the development cohort (Center 1) while the external evaluation was performed on the testing cohort (Center 2).

The development cohort was randomly divided into training and validation sets using a 60%-40% ratio. This split was chosen to preserve a sufficiently large training dataset while maintaining an independent validation set, given the limited sample size. A 10-fold cross-validation strategy was applied to assess the internal validity and robustness of the model and to reduce variance associated with small datasets. For each partition, a model was built using the Multilayer Perceptron node embedded in SPSS Modeler v18.0 (IBM, NY, USA), resulting in a probability prediction for each patient, and each feature was ranked based on its importance in the model. Despite the limited number of retained features, a multilayer perceptron was selected for its ability to model non-linear interactions between radiomic features. Feature reduction was deliberately performed prior to modeling to limit overfitting and improve generalizability. Initial tuning parameters were defined as follows: the softmax activation function, an initial lambda of 5.10^-7^, an initial sigma of 5.10^-5^, an interval center of 0 with an interval offset of +/- 0.5. A single hidden layer was used. Means of the individual probabilities and rankings were provided for the overall evaluation of the model on the training set. The least important feature was then deleted and the models were retrained in each partition. The goal was to reduce the number of features and thus limit overfitting. Maximization of Efficiency (EFF) was used to select the probability threshold and the final model (= MEL-RAD model). The optimal probability threshold was selected by maximizing the Efficiency metric, balancing sensitivity and specificity according to disease prevalence. Efficiency was defined using prevalence (P) as follows: EFF = P × Sensitivity (Se) + (1 − P) × Specificity (Sp).

### 2.6 Statistics

Descriptive statistics were used to characterize the cohort. Quantitative variables were described as median and range. Qualitative variables were expressed as number (n) and percentage (%).

PFS was defined as the time from treatment initiation to progression or death from any cause, whichever occurred first; patients alive and progression-free were censored at the last follow-up. OS was defined as the time from treatment initiation to death from any cause; patients alive were censored at the last follow-up. Survival rates were estimated using the Kaplan–Meier method with 95% confidence intervals (95% CI). Univariable and multivariable analyses were conducted using the log-rank test and Cox proportional hazards model. Hazard ratios (HRs) with their 95% CI were reported. Prognostic factors with a p-value < 0.20 in univariable analysis were included in the multivariable model to assess their interaction and combined effect; this approach was chosen to avoid excluding potentially interacting variables in a limited sample size. Prognostic factors found statistically significant in the multivariable model were subsequently tested in the testing cohort.

The MEL-RAD model was built for the prediction of the 1y-PFS assessed with FDG-PET/CT according to PERCIST criteria (25). The model was thus assessed by receiver operating characteristic: Se, Sp, positive predictive value (PPV) and negative predictive value (NPV). The MEL-RAD model was included in the multivariable analysis to assess inter-feature complementarity.

Validation was performed on the Center 2 using PFS and OS following the same methodology as for the training cohort.

The significance level of the p-value was 0.05. All statistics were performed using MedCalc Statistical software v15.8 (Ostend, Belgium) and SPSS v24.0 (IBM, Armonk, USA).

## 3 Results

### 3.1 Population

Between August 2015 and September 2023, 180 patients with advanced-stage CM who underwent a pre-treatment FDG-PET/CT were identified. Twenty-six patients were excluded from the analysis for one of the following reasons: ICI as second-line treatment (n=20), FDG-PET/CT performed at another center (n=2), or cerebral metastasis only (n=4). A total of 154 patients were followed up and analyzed until September 2024 (**Supplementary Figure 1**).

The main characteristics of the cohort are summarized in **Table 1**. The development cohort consisted of 95 patients (male =50, female =45) with a median age of 67 years (range, 33 to 90) and an ECOG PS of 0/1 in 65/30 patients. The testing cohort consisted of 59 patients (male =36, female =23) with a median age of 66 years (range, 34 to 94) and an ECOG PS of 0/1 in 19/40 patients.

**Table 1:** Characteristics of patients at baseline.

Characteristics	Development cohort Number of patients (n=95)	Testing cohort Number of patients (n=59)
Age (year), median (range)	67 (33–90)	73 (34–94)
Sex (male/female)	50/45	36/23
ECOG PS, n (%)		
0	65 (68.4)	19 (32.2)
1	30 (31.6)	40 (67.8)
Brain metastases, n (%)	16 (16.8)	09 (15.2)
Liver metastases, n (%)	24 (25.3)	14 (23.7)
Metastasis stage, n (%)		
M1a	21 (22.1)	10 (16.9)
M1b	19 (20.0)	12 (20.3)
M1c	39 (41.1)	28 (47.5)
M1d	16 (16.8)	9 (15.3)
Previous treatments		
Surgery	63 (66.3)	36 (61.0)
Radiotherapy	22 (23.2)	13 (22.0)
None	5 (5.3)	3 (5.0)
Treatment, n (%)		
Anti-PD1 (PEMB/NIVO)	63 (66.3)	36 (61.0)
Anti-CTLA4 + Anti-PD1 (IPI+NIVO)	32 (33.7)	23 (39.0)
Corticosteroid treatment, n (%)	25 (26.3)	14 (23.7)
Adverse events with CTCAE 5.0 Grade ≥ 3, n (%)	22 (23.1)	13 (22.0)
Anti-PD1(PEMB/NIVO)	11 (50.0)	02 (15.4)
Anti-CTLA4 + Anti-PD1 (IPI+NIVO)	11 (50.0)	11 (84.6)
Elevated baseline LDH level, n (%)	22 (23.2)	31 (52.5)
BRAF V600 mutation, n (%)	34 (35.8)	13 (22.0)
NRAS mutation, n (%)	38 (40.0)	09 (15.2)

ECOG PS , Eastern Cooperative Oncology Group Performance Status; PEMB, pembrolizumab; NIVO, nivolumab; IPI, ipilimumab; CTCAE, Common Terminology Criteria for Adverse Events; LDH, lactate dehydrogenase; dNLR, derived neutrophil-to-lymphocyte ratio

### 3.2 Outcomes

In the development cohort, median follow-up was 16.8 months (range, 0.6 to 90.9 months). Fifty-eight patients (61.0%) showed disease progression (DP) with a median time to progression of 4.8 months (range, 0.5 to 69.6 months). Of these, 46 patients (48.4%) experienced a 1-year DP with a median time of 4.5 months (range, 0.5 to 11.5 months); and 52 patients (54.7%) died from their CM during the follow-up period, with a median time of 7.4 months (range, 0.5 to 80.1 months).

In the testing cohort, median follow-up was 16.4 months (range, 2.2 to 90.9 months). Thirty-three patients (55.9%) showed DP with a median time of 6.1 months (range, 1.2 to 90.9). Of these, 24 patients (40.7%) experienced 1-year DP, with a median time to progression of 6.1 months (range, 1.2 to 11.7 months); and 28 patients (47.4%) died from their CM during the follow-up period, with a median time of 11.5 months (range, 2.2 to 90.9 months).

### 3.3 Predictive model survival construction on development cohort

**Figure 2:**
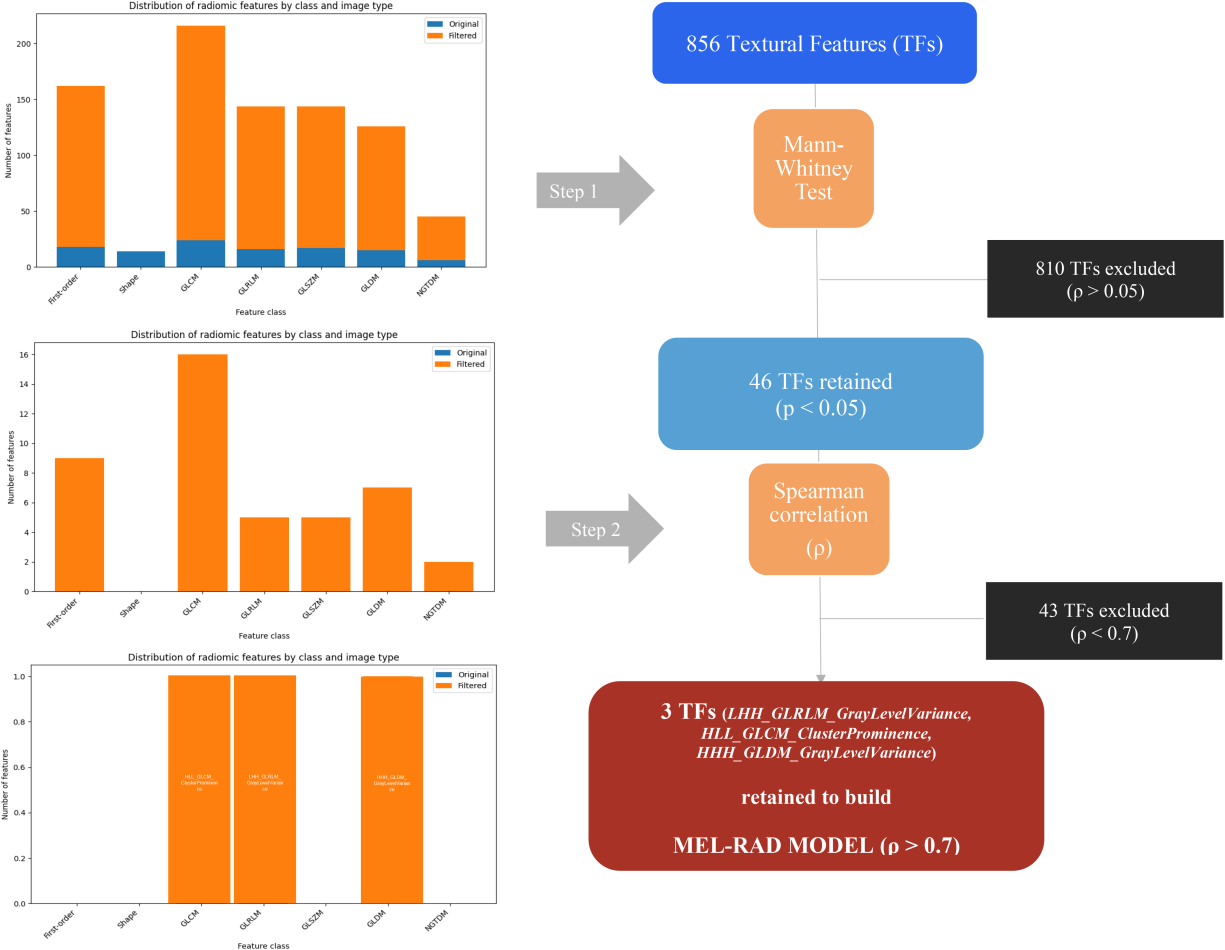
PET textural features selection workflow. (GLCM, Gray Level Co-occurrence Matrix; GLRLM, Gray Level Run Length Matrix; GLSZM, Gray Level Size Zone Matrix; GLDM, Gray Level Dependence Matrix; NGTDM, Neighborhood Gray Tone Difference Matrix).

Of the initially extracted 856 radiomic features (**Supplementary Table 3**), only 46 and then 3 were respectively retained after the first and second steps of the selection workflow, which were 3D wavelet radiomic features (**Figure 2**):

*LHH_GrayLevelRunLengthMatrix_GrayLevelVariance: a* GLRLM-derived feature, quantifying variability of gray-level run lengths,
*HLL_GrayLevelCo-occurrenceMatrix_ClusterProminence:* a GLCM-derived feature, capturing higher-order spatial relationships between gray levels,
*HHH_GrayLevelDependenceMatrix_GrayLevelVariance:* a GLDM-derived feature, quantifying the variance of gray-level dependencies.

The full list is provided as **Supplementary Table 2**.

According to the model building workflow, the best model (MEL-RAD) combining these 3 radiomic features resulted in an area under curve (AUC) of 0.74 (p<0.0001) in the development cohort. Using the 1y-PFS rate as disease prevalence, the efficiency was measured at 50.5% (**Supplementary Figure 2**).

The optimal probability threshold was determined to be 55.0% (C-statistic = 0.63; sensitivity = 93.8%; specificity = 31.9%; PPV = 58.4%; NPV = 83.4%).

#### 3.3.1 Progression free survival

**Figure 3:**
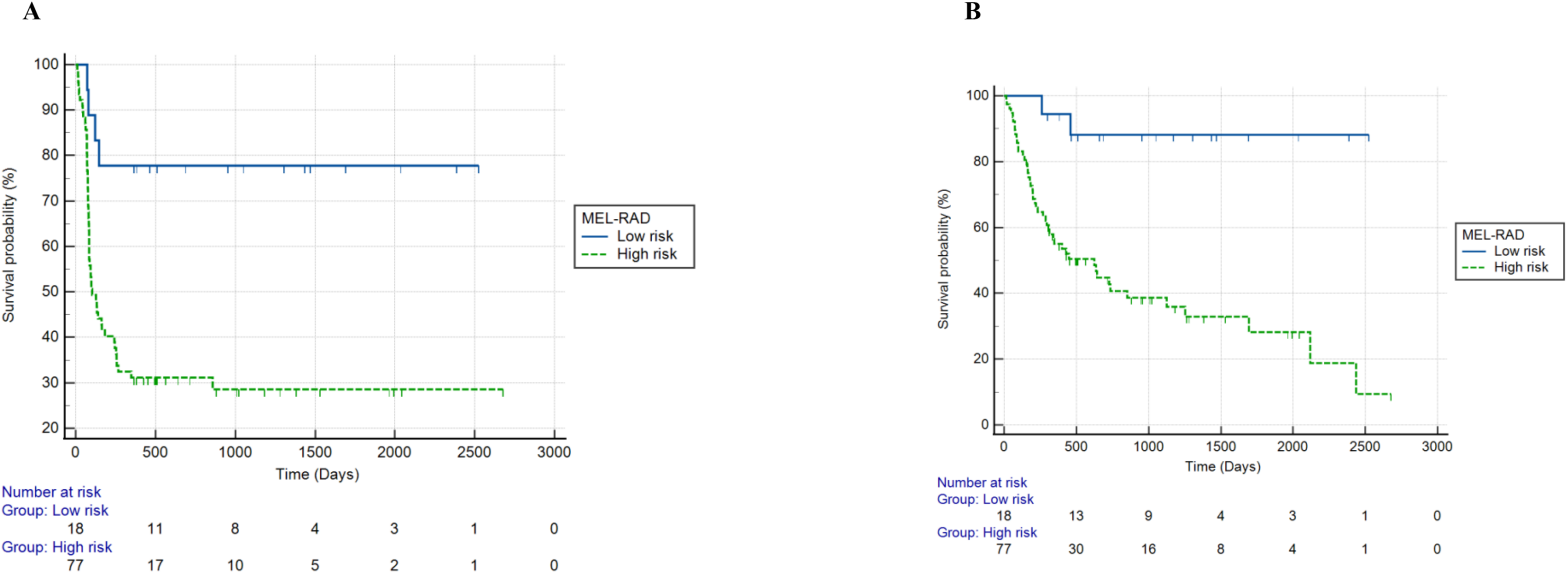
Survival in development cohort as stratified by MEL-RAD model [**(A)** 1-year progression-free survival; **(B)** overall survival].

Patients with a MEL-RAD probability >55%, had a significantly lower PFS, with a HR of 2.73 (95% CI 1.50-4.96, p = 0.0009). The Kaplan-Meier curves are shown in **Figure 3A**.

In both univariable and multivariable analyses, no clinicobiological factors were identified as significant predictors of PFS (**Table 2**).

**Table 2:** Univariable and multivariable analyses of both clinical and biological factors according to progression free survival.

Parameters	Development cohort number of patients (n=95)			
	Univariable		Multivariable	
	HR (95% CI)	*P*-value	HR (95% CI)	*P*-value
Age (year) > 65 vs ≤ 65	0.92 (0.55-1.55)	0.756	–	–
Sex male vs female	0.88 (0.53-1.46)	0.619	–	–
ECOG PS 1 vs 0	1.40 (0.79-2.33)	0.262	–	–
Brain metastases yes, vs no	1.80 (0.95-3.41)	0.071	1.86 (0.97-3.54)	0.068
Hepatic metastases yes, vs no	1.47 (0.82-2.64)	0.092	1. 51 (0.84-2.7)	0.168
Treatment, n (%) Anti-PD1 vs Anti-CTLA4 + Anti-PD1	1.10 (0.61-1.97)	0.694	–	–
Corticosteroids yes, vs no	1.63 (0.96-2.79)	0.063	1.38 (0.76-2.51)	0.287
Elevated baseline LDH yes, vs no	1.46 (0.82-2.60)	0.197	1.34 (0.73-2.54)	0.339

HR, hazard ratio; CI , confidence interval, ECOG PS , Eastern Cooperative Oncology Group Performance Status; LDH, Lactate dehydrogenase, dNLR, derived neutrophil-to-lymphocyte ratio). Significant P-value (< 0.05).

Combining the MEL-RAD model with these clinical parameters did not significantly improve the predictive model.

#### 3.3.2 Overall survival

Patients with a MEL-RAD probability >55% had significantly lower OS (HR = 3.20, 95% CI: 1.70-6.05, p = 0.0003). The corresponding Kaplan-Meier curves are shown in **Figure 3B**.

In both univariable and multivariable analyses, no clinicobiological factors were identified as significant predictors of OS (**Table 3**).

**Table 3:** Univariable and multivariable analyses of both clinical and biological factors according to overall survival.

Parameters	Development cohort number of patients (n=95)			
	Univariable		Multivariable	
	HR (95% CI)	*P*-value	HR (95% CI)	*P*-value
Age (year) > 65 vs ≤ 65	1.03 (0.59-1.79)	0.915	–	–
Sex male vs female	1.02 (0.59-1.75)	0.957	–	–
ECOG PS 1 vs 0	1.30 (0.75-2.28)	0.353	–	–
Brain metastases yes, vs no	1.49 (0.74-2.97)	0.263	–	–
Hepatic metastases yes, vs no	1.61 (0.88-2.95)	0.123	1.60 (0.87-2.93)	0.129
Treatment, n (%) Anti-PD1 vs Anti-CTLA4 + Anti-PD1	1.33 (0.67-2.63)	0.416	–	–
Corticosteroids yes, vs no	1.54 (0.87-2.71)	0.136	1.39 (0.78-2.48)	0.259
Elevated baseline LDH yes, vs no	1.67 (0.91-3.07)	0.097	1.98 (0.94-3.23)	0.089

HR, hazard ratio, CI, confidence interval, ECOG PS , Eastern Cooperative Oncology Group Performance Status, LDH, Lactate dehydrogenase, dNLR, derived neutrophil-to-lymphocyte ratio). Significant P-value (< 0.05).

### 3.4 MEL-RAD model validation on testing cohort

Using a probability threshold of 55%, the MEL-RAD model achieved a C-statistic of 0.62 for 1y-PFS prediction, Se = 95.7%, Sp = 27.8%, VPP = 45.8% and VPN = 90.9%.

#### 3.4.1 Progression free survival

**Figure 4:**
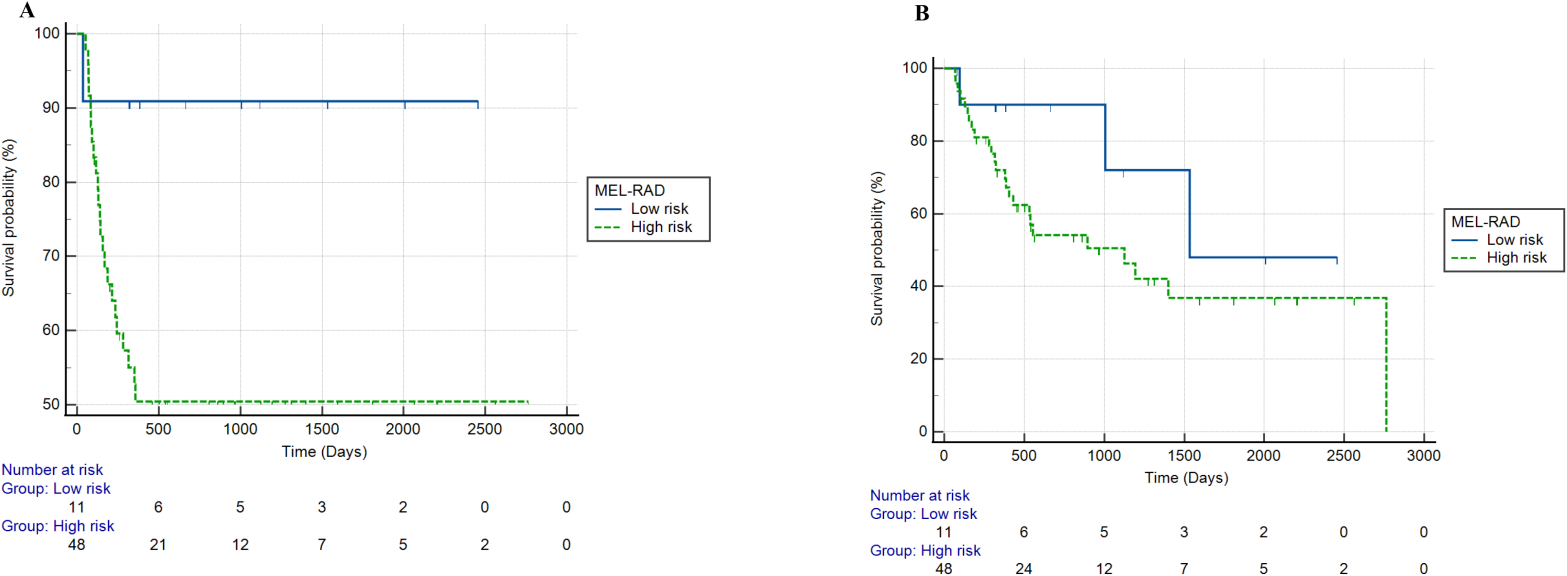
Survival in testing cohort as stratified by MEL-RAD model **(A)** 1-year progression free-survival; **(B)** overall survival).

Patients with a MEL-RAD probability >55% had a significantly lower PFS (HR = 2.73, 95% CI: 1.01-7.38, p = 0.047). The corresponding Kaplan-Meier curves are shown in **Figure 4A**.

#### 3.4.2 Overall survival

Patients with a MEL-RAD probability >55% showed a lower OS, although this difference was not statistically significant (HR = 2.36, 95% CI 0.74-7.54, p = 0.144). The corresponding Kaplan-Meier curves are shown in **Figure 4B**.

## 4 Discussion

To our knowledge, this is the first study to validate a radiomic model based on FDG PET of ICI-treated CM patients in an external cohort.

The cohorts were marked by tumor heterogeneity, including cases with oligometastatic disease, but few other potential confounders, as all patients had an ECOG PS of 0 or 1 and no notable comorbidities. However, differences existed between the two cohorts: the testing cohort had a higher median age, a greater proportion of patients with ECOG PS 1 and a higher prevalence of elevated baseline LDH levels, suggesting a potentially more aggressive disease profile. Despite these differences, other clinical and biological characteristics were comparable between the cohorts.

MEL-RAD model was significantly associated with PFS in both cohorts (development and testing). However, for OS, the model showed a statistically significant association only in the development cohort.

The MEL-RAD model, developed by combining three radiomic features, demonstrated moderate discriminative ability in the development cohort, with an AUC of 0.74 (p < 0.0001). When using the 1y-PFS rate as the disease prevalence, the overall predictive accuracy of the model was estimated at 50.5%, indicating modest performance. An optimal probability threshold of 55% yielded a C-statistic of 0.63, with high Se (93.8%) but low Sp (31.9%). The corresponding PPV and NPV were 58.4% and 83.4%, respectively. External validation of the MEL-RAD model in an independent cohort (testing) confirmed these findings, with a C-statistic of 0.62, Se of 95.7%, Sp of 27.8%, PPV of 45.8%, and NPV of 90.9%. Although the low specificity indicates a substantial rate of false positives, the high Se and elevated NPV suggest that the model is effective in identifying patients who are unlikely to experience disease progression.

Predicting prognosis prior to treatment initiation remains essential for optimizing the management of patients with CM (8, 9, 26). Only three studies have investigated the predictive value of TFs extracted from FDG-PET/CT, but these were based on small sample sizes. Most of the other studies focused primarily on first-order PET indices (27–29). Baseline SUVpeak, MTV, and TLG have also been reported as promising predictors of response to ICI treatment in CM (30), but no clear thresholds have been established to stratify patients into prognostic subgroups. Emerging evidence suggests that MTV may predict survival, although its robustness varies, and further large studies are needed to confirm and validate MTV thresholds for clinical use. Notably, approximately 55% of CM cases show intrinsic resistance to anti-PD-1 inhibitors (with around 40% showing innate resistance to CTLA4 + PD1 inhibitor combination), with 25% of initial responders developing resistance within 2 years (31). In our study, these first-order TFs were excluded from the predictive model due to their limited prognostic value, as they measure global tumor characteristics without analyzing complex relationships between voxels or the internal tumor structures. In contrast, Higher-order TFs, which were found to be significant in our model, provide more complex information reflecting tumor heterogeneity and indicating rapid tumor proliferation (14). The three selected higher-order radiomic features likely capture different aspects of intra-tumoral heterogeneity, which may explain their predictive value. LHH_GLRLM_GrayLevelVariance reflects variability in gray-level run lengths, HLL_GLCM_ClusterProminence emphasizes asymmetry and peakedness in spatial gray-level patterns, and HHH_GLDM_GrayLevelVariance quantifies local intensity dependencies (14, 32–34). Together, these features provide complementary information on tumor texture complexity, potentially underlying the MEL-RAD model’s ability to stratify patient risk. Indeed, despite the limited data available in the literature for these features in PET imaging, GrayLevelVariance measures the dispersion of gray intensities in a given image area. A high variance indicates greater intensity changes, reflecting significant tumor heterogeneity and potential aggressiveness. In Ferrer-Lores’ study on diffuse large B-cell lymphoma, this feature was predictive of a therapeutic response to chemotherapy when its value was low (35). Similarly, Cluster Prominence (CP) is a measure of the asymmetry of a given distribution. High CP values indicate low image symmetry. In medical imaging, low CP values represent a smaller peak for the image gray-level value, and usually, the gray-level difference between the forms is small. This reflects heterogeneity, as the lower the value, the greater the heterogeneity and potential aggressiveness. In the study by Huang et al., this feature was predictive of breast cancer recurrence at 1 year following treatment with chemotherapy, surgery, or radiotherapy (36).

Our model may therefore influence the treatment strategy of metastatic CM under ICI, as it showed superior risk stratification compared to established first-order TFs (9). We proved a risk reduction of almost 20% of DP. Moreover, patients with a MEL-RAD probability >55% could benefit from intensified treatment (anti-PD1 + anti-CTLA4 or anti-LAG3 for PD-L1 <1%). This is consistent with evidence showing improved outcomes and reduced hyperprogression with combination therapy (24). Conversely, those with a MEL-RAD probability ≤55% may be better suited for anti-PD1 monotherapy, which offers fewer adverse events (AEs) (37).

Our clinicobiological results were not statistically significant, likely due to limited statistical power stemming from the relatively small sample size. However, there was a trend toward significance for brain metastases in relation to PFS, which aligns with findings in the literature. In the study by Moyers et al. (38), the presence of brain metastases was associated with shorter PFS compared to their absence. Regarding OS, no significant factors were identified, although elevated baseline LDH levels showed a trend toward significance, consistent with previously published data (39).

We used a robust and rigorous methodology in this radiomic study, which is essential to ensure reproducibility and clinical applicability (14). One of the major challenges in radiomics is therefore to ensure reproducible tumor delineation across studies and different centers and clinical settings, as contour variations can affect the analysis. In our study, we used a validated segmentation technique based on the PERCIST approach for treatment response assessment in delineating the target tumor with a threshold of 1.5 x mean liver SUV in a 3-cm spherical VOI in the right lobe + 2 SD (20). However, this method shares the limitations of absolute SUV-based thresholds, particularly in accounting for intratumoral heterogeneity (40). While the gradient-based method excludes necrotic areas and offers an excellent reproducibility (41), it is not yet widely available with all commercially software (42). To date, there is no consensus on the methodology for defining the metabolic threshold for FDG-PET/CT segmentation (43). Some studies used a fixed SUV threshold for tumor delineation (27, 28, 44), while our study normalized the threshold for each patient using liver uptake as a reference (20). Our semi-automated method combined with a manual correction enables for rapid tumor delineation followed by refinement for greater accuracy and consistency. This hybrid approach improves reproducibility while accommodating complex tumor shapes and heterogeneous regions. However, we acknowledge that our segmentation pipeline does not provide the evaluation of inter-reader variability. Another important challenge in radiomics is the extraction of reliable features from small-volume lesions, which are prone to high noise levels and low signal-to-noise ratios. Extracting radiomic parameters from these small lesions carries the risk of obtaining inaccurate or unreliable features, potentially leading to biased results (45). However, we have demonstrated the feasibility of this approach by using an established feature extraction methodology that has already been externally validated through rigorous testing (46). Our study provides strong evidence that even when integrating small-volume lesions, it is possible to extract meaningful radiomic features that are both robust and reproducible, provided the appropriate methodology is applied. Finally, our strength was to build our prognostic model MEL-RAD from a development cohort and test it in an external validation cohort, which is recognized to improve its applicability in different clinical settings (47). Moreover, the use of the neuroCombat harmonization method (22) in our study minimized the heterogeneity of the multicenter data without compromising the inter-patient variability (48).

Our study has several limitations. First, the cohort is heterogeneous, including several patients with pauci-metastatic disease or with BRAF-mutation or on anti-PD1 monotherapy alone. However, this reflects real-life and current practice. Second, the retrospective nature of the study introduces a risk of selection bias. Third, the follow-up period was approximately 16 months, and although this is sufficiently powered for significant PFS results, a longer-term evaluation would be valuable particularly for OS analysis. Fourth, certain characteristics were not assessed such as the Fitzpatrick skin phototype. Fifth, The MEL-RAD model’s performance profile indicates that, while it may effectively exclude patients at low risk of progression (functioning as a “rule-out” model), its capacity to accurately identify those at high risk remains limited. Finally, the study was conducted in two centers, which is a methodological challenge (49). As mentioned above, we used harmonization procedures between the different machines in order to limit the variability due to the different spatial resolution of the PET systems and image reconstruction methods (50). This ensured the robustness of the model for both internal and external validation.

In conclusion, despite the inherent limitations commonly encountered in predictive modeling (51), our results were highly significant and demonstrated the promising predictive potential of FDG-PET/CT radiomics in metastatic CM patients treated with immunotherapy. This model offers encouraging prospects for therapeutic personalization as a biomarker (52) and lays the groundwork for future multimodal approaches that integrate radiomic features with clinical data in larger cohorts. Such combined strategies hold the potential to ultimately enhance prognostic stratification and guide treatment decisions more effectively in this patient population.

## Data Availability

The original contributions presented in the study are included in the article/**Supplementary Material**. Further inquiries can be directed to the corresponding author/s.

## Supplementary material

Supplementary Table 1 Characteristics of the PET systems at Center 1 and Center 2

Supplementary Table 2 Workflow for extraction and selection of PET texture features (https://doi.org/10.5281/zenodo.15584144)

Supplementary Table 3 Distribution of extracted radiomic features by feature class

Supplementary Figure 1 Flowchart of the study profile

Supplementary Figure 2 Efficiency of the MEL-RAD model
