# Supplementary material for: 18F-FDG PET radiomic analysis to predict outcomes in metastatic melanoma treated with immune checkpoint inhibitors
Source: Front Immunol. 2026 Feb 25;17:1642620. doi: 10.3389/fimmu.2026.1642620 (PMC12975435; doi:10.3389/fimmu.2026.1642620)
Supplement: Supplementary Figure 1 — Flowchart of the study profile [file DataSheet1.docx]

**Supplemental data:**

**Tables:**

- **S.Table 1:** Characteristics of the PET systems at Center 1 and Center 2
- **S.Table 2:** Workflow for extraction and selection of PET texture features

(<https://doi.org/10.5281/zenodo.15584144>)

- **S.Table 3:** Distribution of extracted radiomic features by feature class

**Figures:**

- **S.Figure 1***:* Flowchart of the study profile
- **S.Figure 2*:*** Efficiency of the MEL-RAD model

**S.Table 1:** Characteristics of the PET systems at Center 1 and Center 2.

|  |  | | **PET** | | | | | | | **CT** | | | | |
| --- | --- | --- | --- | --- | --- | --- | --- | --- | --- | --- | --- | --- | --- | --- |
|  |  | | **FDG dosage**  **(MBq/kg)** | **Field of view**  **(mm)** | **Voxel resolution**  **(mm)** | **Size of**  **reconstruction transaxial matrix**  **(voxel)** | **Reconstruction**  **method** | **Collimation**  **(mm)** | **Pitch** | | **Tube voltage**  **(kV)** | **Effective tube current**  **(mAs)** | **Number**  **of**  **CT**  **slices** |  |
| **Center 1**  (n=98) | **Siemens Biograph mCT** | 3 | | 700 | 4.07 x 4.07 x 2 | 200 x 200 | OSEM 3D  PSF + ToF  2 iterations,  21 subsets with a 2 mm Gaussian filter | 64 x 1.2 | 1 | | 120 | 80 | 64 |  |
|  | **Siemens**  **Biograph Vision 600** | 3 | | 700 | 1.65 x 1.65 x 1.65 | 440 x 440 | OSEM 3D  PSF + ToF  3 iterations,  5 subsets with a 2 mm Gaussian filter | 64 x 1.2 | 1 | | 120 | 80 | 64 |  |
| **Center 2**  (n=71) | **Siemens Biograph Horizon** | 2 | | 700 | 2.89 x 2.89 x 2.02 | 256 x 256 | OSEM 3D  PSF + ToF  2 iterations,  18 subsets with a 2 mm Gaussian filter | 16 x 1.2 | 1.25 | | 110 | 100 | 16 |  |
|  | **Siemens**  **Biograph**  **Vision 450** | 2 | | 700 | 1.65 x 1.65 x 1.65 | 440 x 440 | OSEM 3D  PSF + ToF  ± SubtlePET^TM^ IA  3 iterations,  5 subsets with a 2 mm Gaussian filter | 64 x 0.6 | 1.25 | | 120 | 110 | 64 |  |

*(Abbreviations: OSEM=ordered subset expectation maximization; PSF=point spread function; ToF=time of flight).*

**S.Table 2:** Workflow for extraction and selection of PET texture features.

(<https://doi.org/10.5281/zenodo.15584144>).

**S.Table 3:** Distribution of extracted radiomic features by feature class

| **Feature class** | **Number of features (%)** |
| --- | --- |
| First-order | 165 (19.3) |
| Shape | 16 (1.9) |
| Gray Level Co-occurrence Matrix (GLCM) | 216 (25.2) |
| Gray Level Run Length Matrix (GLRLM) | 144 (16.8) |
| Gray Level Size Zone Matrix (GLSZM) | 144 (16.8) |
| Gray Level Dependence Matrix (GLDM) | 126 (14.7) |
| Neighborhood Gray Tone Difference Matrix (NGTDM) | 45 (5.3) |

Testing cohort

(n=59)

ICI as 2^nd^ line treatment (n=20)

PET/CT at a different center (n=2) (n=26)

No extracerebral lesion (n=4)

Patients from Center 1

(n=109)

Development cohort

(n=95)

Patients from Center 2

(n=71)

**S.Figure 1*:*** Flowchart of the study profile.


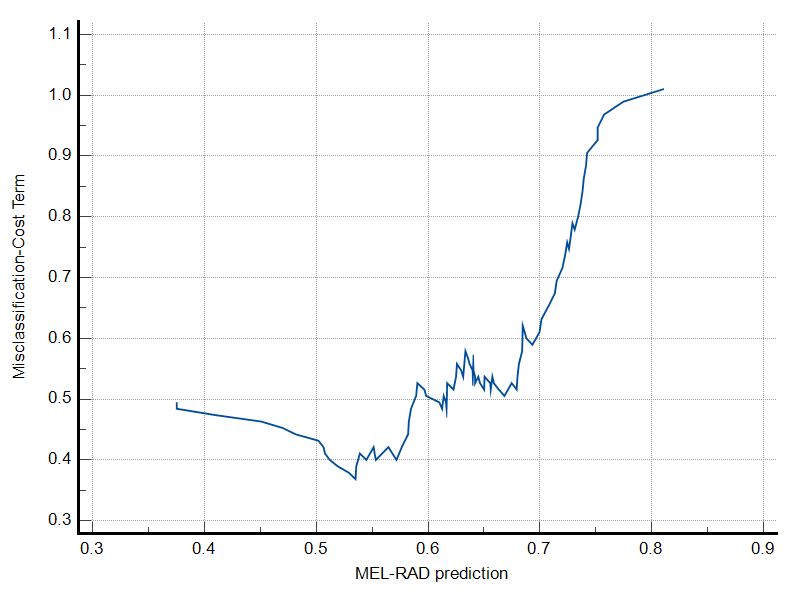


**S.Figure 2*:*** Efficiency of MEL-RAD model
